# Key Ecological Factors Influencing Foraging Site Selection During Different Breeding Stages of the Endangered Scaly‐Sided Merganser in Northeast China

**DOI:** 10.1002/ece3.71011

**Published:** 2025-03-06

**Authors:** Shengxian He, Wenyu Xu, Facai Yang, Chen Zhang, Keping Sun, Longcheng Fan, Longru Jin, Haitao Wang

**Affiliations:** ^1^ Jilin Engineering Laboratory for Avian Ecology and Conservation Genetics, School of Life Sciences Northeast Normal University Changchun China; ^2^ Jilin Provincial Key Laboratory of Animal Resource Conservation and Utilization Northeast Normal University Changchun China

**Keywords:** breeding period, Changbai Mountains, foraging site selection, *Mergus squamatus*

## Abstract

Studies on foraging site selection during the breeding period of waterbirds can identify key ecological factors, providing a scientific foundation for their conservation and habitat management. The Scaly‐sided Merganser ( *Mergus squamatus* ) is a globally endangered species and serves as an indicator species in submontane valleys. However, research on the key ecological factors influencing foraging site selection at different breeding stages remains limited. In this study, ecological variables were collected from 226 sites in the Changbai Mountains, Northeast China, including 115 foraging sites and 111 control sites, across various breeding stages of *M. squamatus* . The analysis focused on foraging site selection during the egg‐laying, incubation, and brooding periods of *M. squamatus* . The results indicated that concealment (C) was the key ecological factor influencing foraging site selection for *M. squamatus* during the egg‐laying period. During incubation, the distance to potential nesting sites (DPNS) and the distance from buildings (DB) emerged as significant factors. In contrast, the brooding period highlighted the importance of the borderland ratio (BR) and heartland ratio (HR). These variations in key ecological factors at different breeding stages are likely due to the distinct physiological and behavioral requirements of *M. squamatus* at each stage.

## Graphical abstract

Key ecological factors influencing the selection of foraging sites by the Scaly‐Sided Merganser across different breeding stages.

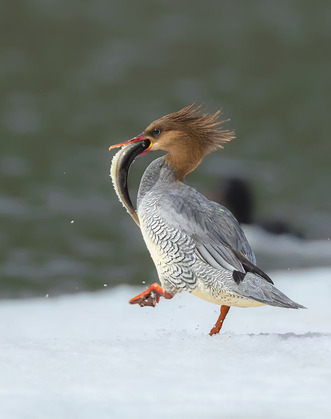

## 1 Introduction

Foraging habitat selection refers to the non‐random use of different foraging sites by individual animals, which affects their survival and adaptability (Van Beest et al. 2010). Factors influencing this selection include food availability, habitat characteristics, concealment conditions, predation risk, human disturbance, and so forth (Jakubas et al. 2007; Rayner et al. 2010; Deshwal et al. 2022; Cheng 2022; Kovinka et al. 2023). For instance, food abundance on beaches influences foraging site selection by the Hooded plover ( *Thinornis rubricollis* ) (Cuttriss et al. 2015); Some fruit‐eating birds trade‐off between food resources and predation risk at migratory stopover sites, choosing foraging sites accordingly (McCabe and Olsen 2015). Therefore, studying animal foraging site selection can reveal how animals access food and make behavioral decisions to mitigate risks (Michelangeli et al. 2015; Deshwal et al. 2022), as well as identify key factors influencing foraging habitat selection and provide a scientific basis for habitat protection and management (Schmidt 1999; Ripari et al. 2022).

A multi‐scale study of foraging site selection can comprehensively reveal the dynamic distribution patterns and behavioral strategies (Dai et al. 2007; Rather et al. 2020). At different spatial scales, for instance, during the wintering period, the Red‐crowned crane ( *Grus japonensis* ) prefers sites with higher land use rates at a regional scale, while at the landscape scale, it favors areas with a greater percentage of reeds and bulrushes (Wang 2021). On the time scale, the Crested Ibis ( *Nipponia nippon* ) forages in areas with minimal human interference during winter, prefers shallow soil during the breeding period, and feeds in shallow areas of larger, clearer waters during the wandering period (Song 2012). Additionally, many waterbirds actively adjust their selection of foraging sites based on seasonal variations or changes in life‐history stages to enhance suitability and optimize foraging efficiency (Zheng et al. 2015; Fan et al. 2020; Yu et al. 2020). Therefore, studying foraging site selection across different time and spatial scales can provide a better understanding of the ecological needs of birds (Jin and Qin 2020; Nugent et al. 2022).

The Scaly‐sided Merganser ( *Mergus squamatus* , SSME) is a globally endangered species with a very small population size, estimated at only 2400–4500 mature individuals. It primarily feeds on fish (Zhao and Piao 1998) and is closely associated with riparian habitats that feature rich aquatic life. The species also serve as an indicator species in submontane valleys (Xu, Solovyeva, et al. 2021). The breeding populations of SSME are primarily distributed in Southeast Russia, North Korea, and Northeast China. Major threats of SSME within the breeding range include riverbank logging, illegal hunting, disturbance from motorboats, river pollution, natural predators, entanglement in fishing nets, and dam construction (BirdLife International 2024). Since the 1970s, researchers have explored various aspects of the SSME, including population size and distribution (Zeng 2014; Zeng, Zhang, et al. 2015; Xu, Wang, et al. 2021), diet (Zhao and Piao 1998), behavioral ecology (Wang et al. 2020; Liu, Chen, et al. 2023), breeding ecology (Liu et al. 2014); Liu, Liu, et al. 2023, and habitat evaluation (Xu 2023). Research on overwintering habitat selection of the SSME revealed that factors such as gravel beach area, fish resources, and water depth significantly influence their habitat selection (Zeng, Shi, et al. 2015). Habitat selection for nest sites during the breeding period indicates that the SSME prefers areas with large tree diameters at breast height, high concealment, tall tree height, and dense shrub cover (Guan 2009). Xu (2023) found that SSME abundance increases with the proportion of forest, river curvature, river width, and river depth. Despite these insights into habitat selection, the foraging sites of the SSME during different breeding stages remain largely unknown.

In this study, we observed and recorded the foraging site of SSME at different breeding stages and measured various ecological factors across different environments during the breeding period. Based on previous studies indicating that habitat selection by waterbirds may vary at different breeding stages (Jiao 2016), we hypothesized that key ecological factors would influence foraging site selection differently at each stage of breeding. Our goal was to explore how SSMEs adjust their foraging sites throughout different breeding stages and identify the key ecological factors that impact foraging site selection.

## 2 Materials and Methods

### 2.1 Study Area

**FIGURE 1:**
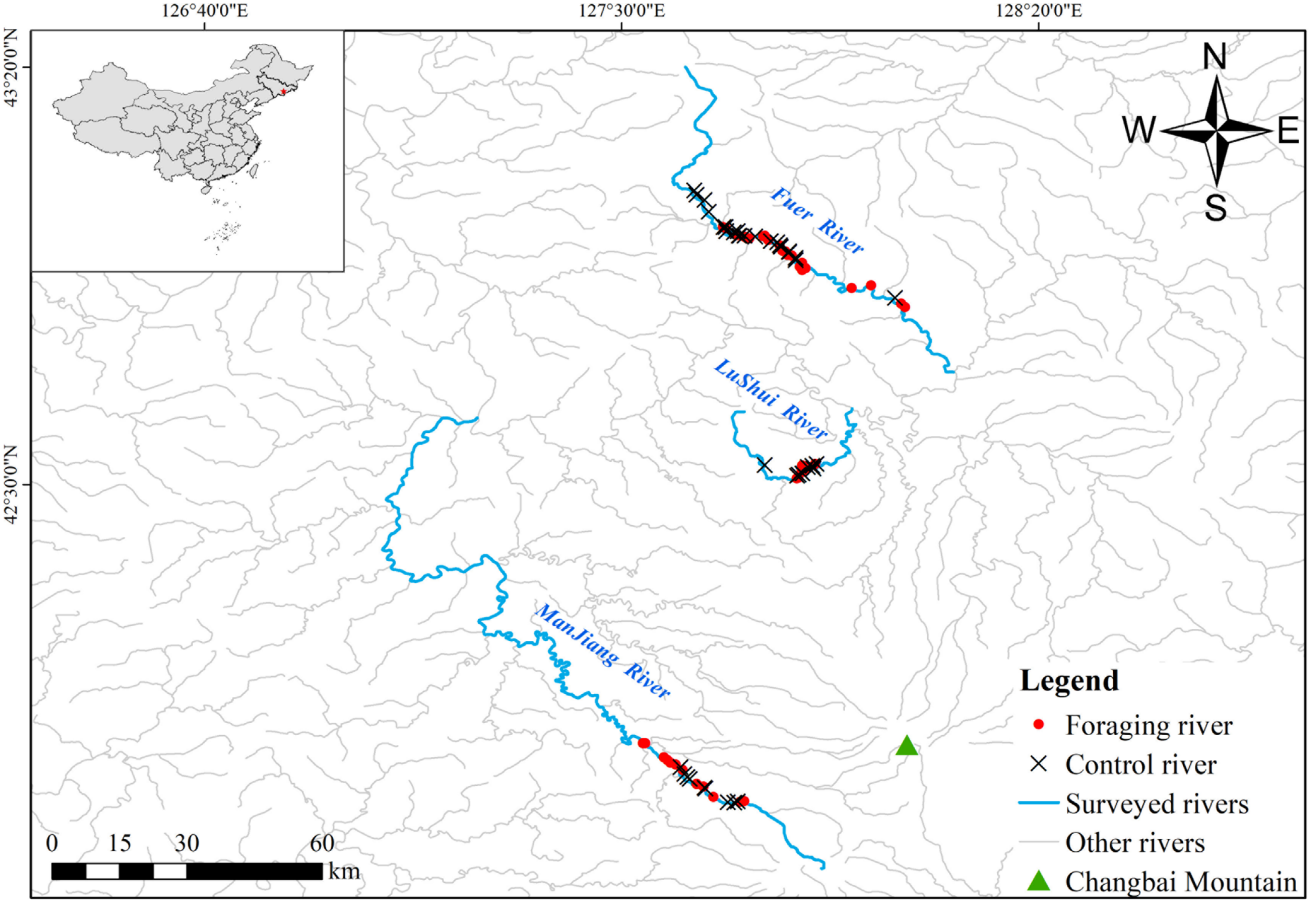
The study area and the sampled river sections.

The study area is located in the Changbai Mountain region in the eastern part of Jilin Province, Northeast China (41°37′‐43°57′ N, 127°6′‐128°20′ E; Figure 1). Approximately 155–170 breeding pairs are found in this area, representing about 90% of the breeding population in China (Xu 2023). It includes the administrative regions of Fusong County, Baishan City, and Dunhua City within the Yanbian Korean Autonomous Prefecture. The region has a temperate continental monsoon climate, characterized by a lack of a distinct hot season. The average annual temperature is approximately 3.8°C, with annual precipitation around 680 mm (Xu et al. 2022).

The region is characterized by numerous rivers and streams that feed into three major water systems: the Songhua River, Yalu River, and Tumen River. It is also regarded as a primary breeding ground for SSME in the Changbai Mountains, supporting over 300 individuals annually (Solovyeva et al. 2014). This area lies within the northeast Palearctic region, known for its rich biodiversity. Records document 67 mammal species across 7 orders, 20 families, and 44 genera, as well as 264 bird species across 17 orders and 46 families (Xu 2023).

### 2.2 Bird Observation Methods

The field survey was conducted during the breeding period of 2023–2024, from mid‐March to early July. Our focus was on the Lushui River, Manjiang River, and Fuer River (Figure 1). The methodology involved using binoculars (Leica, 10 × 42) alongside field observation techniques, including spot monitoring and the feces observation method. In difficult survey locations, a drone (Phantom 2 Vision+) was used to enhance our observations. Upon locating the SSME, we used the focal animal observation method (Bibby et al. 1998) and the full event recording method (Lehne 1996; Wu 2019) to study its behavior. To ensure the accuracy of SSME counts, only individuals that were not flying (e.g., foraging in water or resting on land) were included (Xu, Solovyeva, et al. 2021). Foraging sites were identified based on observed foraging behaviors, which typically lasted between 3 and 15 min (Liu, Chen, et al. 2023).

### 2.3 Site Measurements

After the SSME finished foraging and departed the site, we used a handheld GPS device (Gamma GPSMAP 66 s) to record the location. We recorded tree species (TS), total number of trees (TT), and shrub density (SD) (Li 2023) within a 20‐m radius around the foraging site, as the SSME typically foraged within this range based on our field observations. Subsequently, we used a laser rangefinder (DLX‐DEME16X) to measure tree height and calculate the average height of trees (TH). A meter tape (Deli; 0–5 m) was used to measure the maximum diameter at breast height of trees (MDBH), as well as the average height of shrubs (SH) and herbs (HH). Herb canopy cover (HCC) was estimated using the branch and leaf cover method (Li 2023). Relative concealment (C) was assessed on a five‐point scale (0–4), where 0 indicates no vegetation cover in any direction and 4 indicates complete obscuration in all directions. This assessment was conducted from four directions (east, west, south, and north) at a distance of 20 m from the foraging site. We measured river depth (RD) (Dietrich and Smith 1984), river width (RW) (Jowett and Richardson 1995), water velocity (WV) (Yu et al. 2024), and turbidity (T) at the foraging site, as well as 10 m upstream and downstream, using a metal folding telescopic pole, a laser rangefinder (DLX‐DEME16X), a portable water velocity meter (LS300A), and a portable turbidimeter (ZD‐2A), respectively. The mean values for RD, RW, WV, and T were then calculated. Additionally, we used ArcGIS 10.2 to analyze a 0.5 m resolution satellite map from Google Earth to determine road distance (DR), distance from buildings (DB), distance to potential nesting sites (DPNS), heartland ratio (HR) of the river, and borderland ratio (BR) (Weslati et al. 2020). The HR and BR represent the proportions of borderland within 100 m above and below the river from the foraging sites (Meixler and Bain 2010). Following the method of comparing available versus used sites (Jones 2001; Manly et al. 2007), we randomly selected river sections at least 500 m from the foraging site as control sites and measured the same variables in these control sections.

### 2.4 Breeding Period Division and Sample Size

The study investigated the occupancy of SSME nesting sites within a 0–2 km radius of their foraging sites, based on preliminary findings indicating that foraging sites were typically within this range. When SSMEs were away from their nest boxes, a solar‐powered miniature camera system (SINLIHE MF‐08‐1) was installed in both occupied natural tree holes and artificial nest boxes. The video footage was periodically reviewed via wireless transmission to document the date of the first egg‐laying, as well as incubation and brooding periods. To clarify the different breeding stages, we combined observations of foraging behaviors and the presence or absence of chicks with data from the literature (Lu et al. 2021). The breeding period was divided into three stages: egg‐laying (March 20 to April 15, 2023; March 30 to April 25, 2024), incubation (April 25 to May 20, 2023; May 6 to June 1, 2024), and brooding (May 25 to June 30, 2023; June 6 to July 10, 2024). Individuals for which the breeding stages could not be precisely determined using the aforementioned methods were excluded from the analysis. In total, 226 sites were measured, including 42 foraging sites and 44 control sites during the egg‐laying period, 37 foraging sites and 32 control sites during the incubation period, and 36 foraging sites and 35 control sites during the brooding period (Figure 1).

### 2.5 Data Analysis

Among the 18 ecological factor variables, the Kolmogorov–Smirnov test was used to assess the normality of continuous variables. For normally distributed variables, Student's *t*‐test was used to compare their means between foraging and control sites. For non‐normally distributed variables, the Mann–Whitney *U*‐test was applied (Liu 2023). The Chi‐Squared Test (CST) was used for categorical variables (Li 2023). Factors showing statistical significance (*p* < 0.05) were included in subsequent analyses (Bateson and Martin 2021). Spearman's rank correlation test and variance inflation factor (VIF) analysis (Kajtoch et al. 2014) were employed to examine correlations among variables at different breeding stages. These variables included C, DB, and WV during the egg‐laying stage; DPNS during incubation; and SD, DB, and RW during the brooding stage. Variables with a VIF > 4 were excluded (Li 2023). Correlation coefficients |r| ≤ 0.75 and VIF < 4 were tested for nine variables during the egg‐laying period, six variables during incubation, and nine variables during the brooding period. The retained variables met these criteria (Jara et al. 2020; Zuur et al. 2009). Prior to analysis, the characteristic variables were standardized using Z‐score normalization. The effects on foraging were then analyzed using logistic regression with a generalized linear mixed model (GLMM), implemented in the lme4 package in R. This analysis was conducted for each of the three breeding stages.

Key ecological factors influencing foraging site selection were modeled using logistic regression. In this model, river number and year were treated as random effects, while the foraging and control site variables were treated as independent variables. The dependent variable indicated whether foraging occurred or not (Xu 2023). Model selection was performed using the dredge function from the boral package to screen models based on the corrected Akaike information criterion (AICc) and models with ΔAICc ≤ 2 were selected. The model with the smallest ΔAICc value was considered the best (Symonds and Moussalli 2011). If multiple models had ΔAICc ≤ 2, they were averaged using the “MuMIn” package in R (Stoica et al. 2004).

Data are presented as mean ± standard deviation (SD). All statistical analyses were performed using SPSS 27.0 and R 4.1.2.

## 3 Results

Over the course of 204 days in the field, we surveyed more than 180 km of rivers by walking and using a drone. During this period, we observed and counted 153 individuals of SSME: 39 in the Lushui River, 63 in the Manjiang River, and 51 in the Fuer River.

### 3.1 Key Ecological Factors Influencing Foraging Site Selection During the Egg‐Laying Period

**FIGURE 2:**
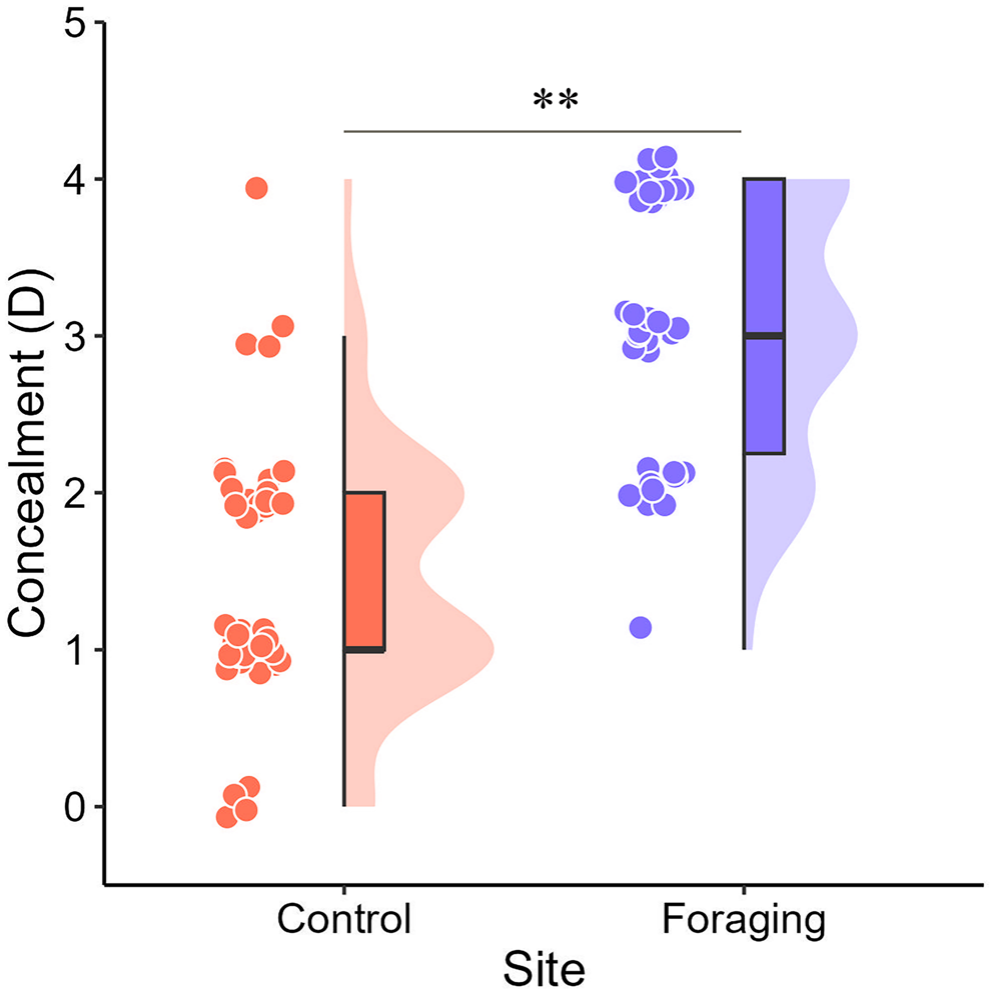
Comparison of foraging and control sites for C during the egg‐laying period of SSME. Circles represent numerical values, the median lines indicate the median value, and the upper and lower limits represent the 25th percentiles (high and low values, respectively). **: *p* < 0.01, as shown below.

During the egg‐laying period, significant differences were observed in MDBH, SD, C, HR, BR, DB, RD, WV, and T, while no significant differences were found in TS, TT, TH, SH, HH, HCC, DR, DPNS, and RW (Table 1). Logistic regression was used to analyze several variables, including MDBH, SD, HR, BR, C, DB, RD, WV, and T. Model‐I, which included only the variable C, showed the highest interpretability (Table 2). Foraging sites were significantly more cryptic than control sites (Figure 2). Model averaging results indicated that the probability of selecting a foraging site during the egg‐laying period of SSME increased with the degree of C (*β* = 1.67; *p* < 0.01) (Table 3).

**TABLE 1:** Differences in habitat characteristics between foraging and control sites during the egg‐laying period of SSME.

Variables	Foraging	Control	Statistics			*p*
			*t*	*Z*	*χ* ^*2*^	
TS (*n*)	14.00 ± 2.45	13.34 ± 2.53		1.23		0.22
TT (*n*)	129.71 ± 34.47	126.61 ± 34.15		0.40		0.69
TH (m)	26.93 ± 7.28	25.29 ± 6.23	1.12			0.27
MDBH (cm)	41.70 ± 6.54	36.86 ± 5.97	3.59			< 0.01**
SH (m)	3.15 ± 0.62	3.15 ± 0.83	0.01			0.99
SD (%)	29.84 ± 7.14	24.98 ± 7.76	3.01			< 0.01**
HH (cm)	20.15 ± 4.24	20.60 ± 4.20	0.49			0.63
HCC (%)	22.96 ± 5.79	23.25 ± 6.48	0.22			0.83
C (D)	3.07 ± 0.84	1.43 ± 0. 84			8.76	< 0.01**
DR (m)	653.77 ± 501.59	913.46 ± 499.36		2.41		0.06
HR (%)	9.64 ± 4.64	5.39 ± 1.51	5.76			< 0.01**
BR (%)	14.17 ± 4.40	6.79 ± 3.23	8.90			< 0.01**
DB (m)	3057.15 ± 859.09	1189.18 ± 489.60		12.46		< 0.01**
DPNS (m)	1402.43 ± 363.94	1463.35 ± 262.41	0.89			0.37
RD (m)	1.21 ± 0.41	1.01 ± 0.52	1.05			0.04*
RW (m)	23.71 ± 8.18	27.64 ± 13.74	1.60			0.11
WV (m/s)	0.36 ± 0.07	0.48 ± 0.23		3.15		< 0.01**
T (NTU)	3.62 ± 0.61	6.03 ± 1.18	11.82			< 0.01*

*Note:* * represents *p* < 0.05 and ** represents *p* < 0.01.

**TABLE 2:** Results of AIC‐based model selection identifying the main factors that influence the foraging sites and control sites during the egg‐laying period of SSME.

No	Model	df	LogLik	AICC	△AICc	ωi
1	C	1	−45.62	61.54	0.00	0.23
2	BR + C	3	−48.77	61.85	0.31	0.19
3	HR + DB	2	−46.25	62.41	0.87	0.17
4	RD + SD + DB	4	−46.64	62.77	1.23	0.15
5	MTBH+T + WV	3	−50.14	63.02	1.48	0.13
6	BR + HR + T	5	−57.35	63.50	1.96	0.09

*Note:* For each model, the number of parameters (df), loglikelihood (LogLik), corrected Akaike information criterion (AICc), difference in AICc between the given model and best model (ΔAICc), and model weight (ωi) are provided. Only models with ΔAICc ≤ 2 are listed in the table, as shown below.

**TABLE 3:** Key factors influencing foraging site selection during the egg‐laying period of SSME after model averaging.

Parameter	Estimate	*p*	95% confidence interval
MDBH	0.11	0.25	0.026 to 0.238
SD	0.08	0.16	0.021 to 0.162
C	1.67	< 0.01**	0.120 to 3.280
HR	0.35	0.09	0.146 to 0.579
BR	0.43	0.07	0.173 to 0.655
DB	0.59	0.14	0.062 to 1.098
RD	0.53	0.32	0.119 to 0.931
WV	0.36	0.06	0.133 to 0.437
T	0.17	0.18	0.044 to 0.267

*Note:* ** represents *p* < 0.01.

### 3.2 Key Ecological Factors Influencing Foraging Site Selection During the Incubation Period

**FIGURE 3:**
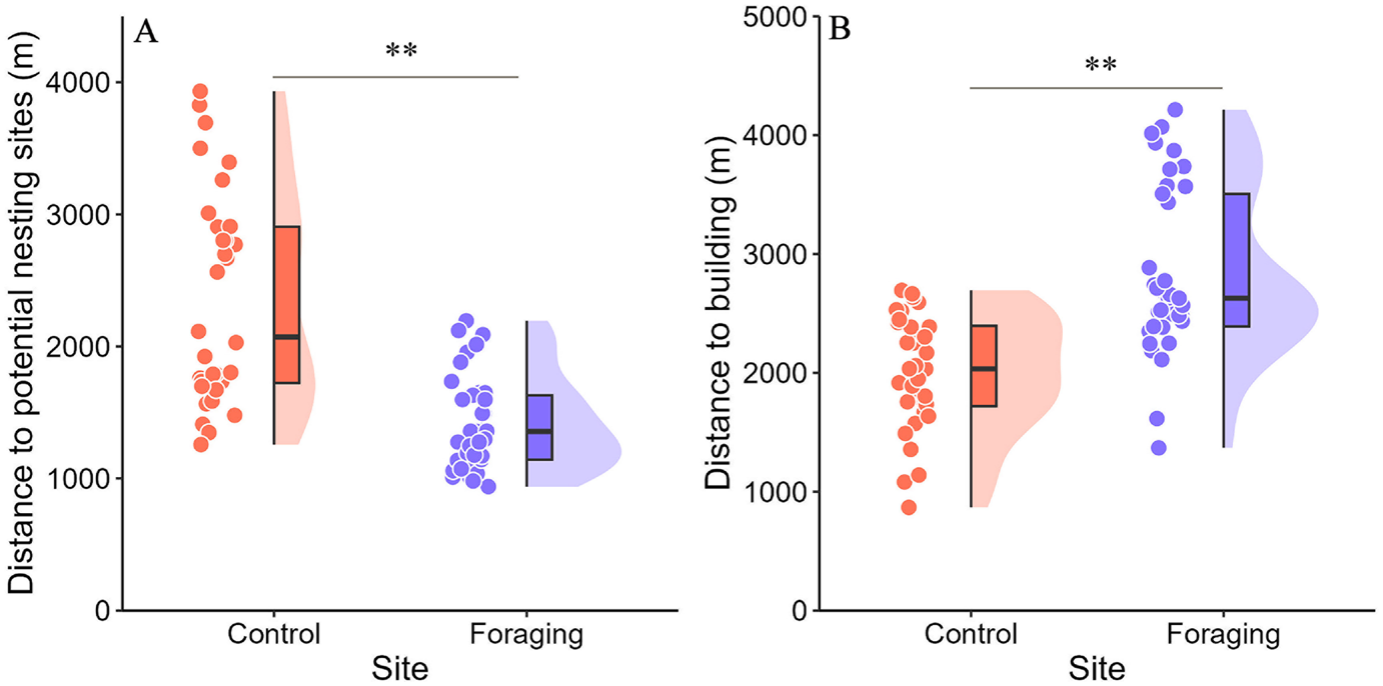
Comparison of DPNS (A) and DB (B) for foraging sites during the incubation period of SSME with control sites.

During the incubation period, significant differences were observed in MDBH, DB, DPNS, RD, WV, and T, while TS, TT, TH, SH, SD, HH, HCC, C, DR, HR, BR, and RW showed no significant differences (Table 4). Model‐I provided the best interpretation rate (Table 5) among the variables with significant differences: MDBH, DB, DPNS, RD, WV, and T. The key influencing factors were DPNS and DB. DPNS was lower in foraging sites compared to the control sites (Figure 3A), whereas DB was higher in foraging sites than in control sites (Figure 3B). Model‐averaged results indicated that the probability of selecting foraging sites during incubation increased with decreasing DPNS (*β* = 0.83; *p* < 0.01) and increasing DB (*β* = 1.38; *p* < 0.01) in SSME (Table 6).

**TABLE 4:** Differences in foraging and control site characteristics during the incubation period of SSME.

Variables	Foraging	Control	Statistics			*p*
			*t*	*Z*	*χ* ^*2*^	
TS (*n*)	15.00 ± 2.39	14.56 ± 2.11	0.80			0.43
TT (*n*)	139.86 ± 23.72	138.31 ± 25.57	0.26			0.80
TH (m)	35.89 ± 5.49	34.25 ± 7.94		1.01		0.32
MDBH (cm)	45.08 ± 6.82	39.81 ± 6.54	3.26			< 0.01**
SH (m)	2.89 ± 0.62	2.66 ± 0.57	1.65			0.10
SD (%)	30.50 ± 5.51	28.15 ± 6.06	1.68			0.10
HH (cm)	22.48 ± 3.90	21.91 ± 4.65	0.55			0.59
HCC (%)	28.95 ± 9.36	26.36 ± 7.37	1.27			0.21
C (D)	2.38 ± 1.23	2.22 ± 1.04			0.58	0.57
DR (m)	963.37 ± 689.73	1148.67 ± 788.86	1.04			0.30
HR (%)	7.54 ± 1.78	7.69 ± 2.05	0.33			0.75
BR (%)	7.33 ± 1.97	7.17 ± 1.34	0.39			0.70
DB (m)	2830.81 ± 701.30	2001.00 ± 487.37	5.62			< 0.01**
DPNS (m)	1415.69 ± 351.67	2357.19 ± 802.34		6.46		< 0.01**
RD (m)	1.95 ± 0.53	1.41 ± 0.47	4.48			< 0.01**
RW (m)	42.81 ± 12.79	38.07 ± 17.86	2.63			0.06
WV (m/s)	0.35 ± 0.09	0.48 ± 0.12	5.12			< 0.01**
T (NTU)	2.82 ± 0.75	6.75 ± 0.88	19.93			< 0.01**

*Note:* ** represents *p* < 0.01.

**TABLE 5:** Results of AIC‐based model selection highlight the main factors influencing foraging and control sites during the incubation period of SSME.

No	Model	df	LogLik	AICC	△AICc	ωi
1	DB + DPNS	4	−67.04	31.24	0.00	0.31
2	DPNS+WV	2	−68.19	32.37	1.13	0.22
3	RD + WV + DB	4	−69.67	33.00	1.76	0.16
4	RD + WV + T	5	−68.06	33.37	2.13	0.12
5	RD + WV + MDBH	3	−76.56	33.69	2.45	0.11

**TABLE 6:** Key factors influencing foraging site selection during the incubation period of SSME after model averaging.

Parameter	Estimate	*p*	95% confidence interval
MDBH	0.24	0.15	0.015 to 0.429
DPNS	0.83	< 0.01**	0.064 to 1.588
RD	0.45	0.17	0.043 to 0.788
DB	1.38	< 0.01**	0.347 to 2.356
WV	0.18	0.12	0.017 to 0.328
T	0.42	0.28	0.065 to 0.698

*Note:* ** represents *p* < 0.01.

### 3.3 Key Ecological Factors Influencing Foraging Site Selection During the Brooding Period

**FIGURE 4:**
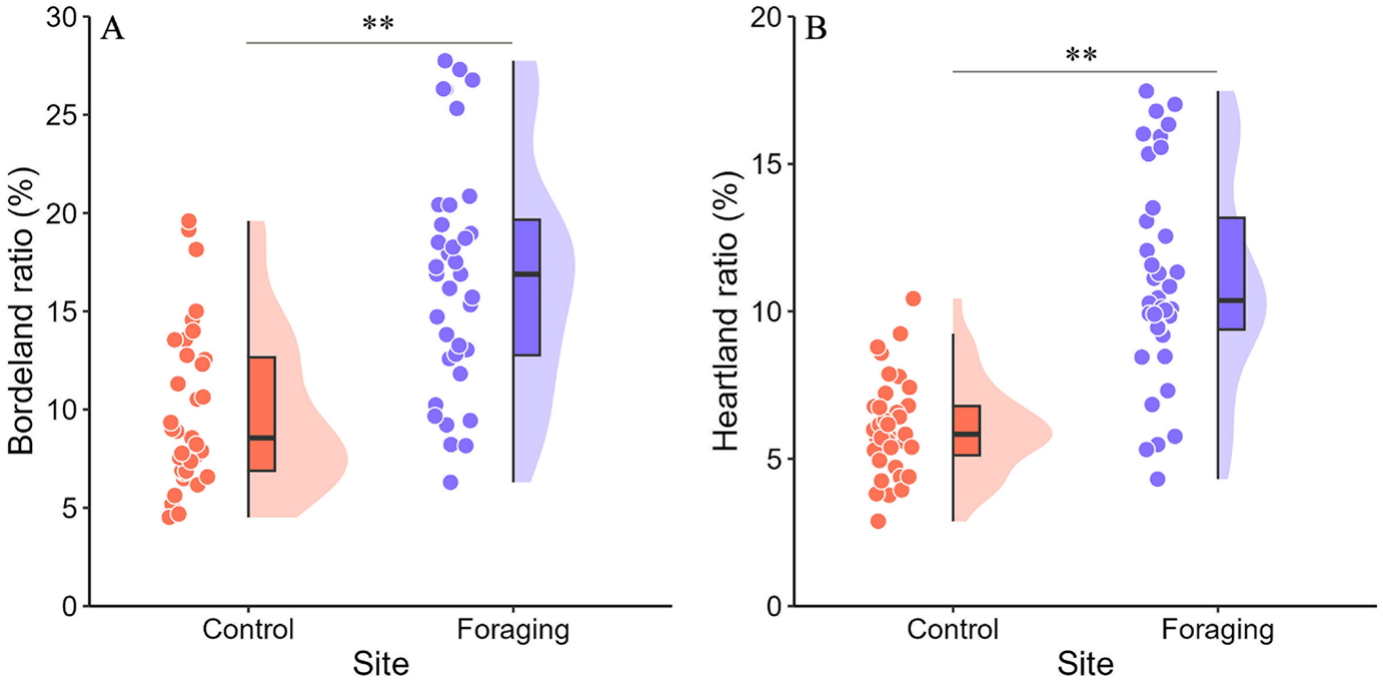
Comparison of BR (A) and HR (B) between the foraging and control sites during the brooding period of SSME.

During the brooding period, significant differences were observed in SD, RD, RW, HR, BR, DR, DB, WV, and T, while TS, TT, TH, MDBH, SH, HH, HC, C, and DPNS were not significant (Table 7). Model‐I demonstrated the best interpretation power (Table 8) based on logistic regression analysis of SD, DR, DB, HR, BR, RW, RD, WV, and T. The key influencing factors were BR and HR. Both BR and HR were significantly higher in the foraging sites compared to the control sites (Figure 4A,B). During the brooding period, the probability of selecting foraging sites increased with larger BR (*β* = 0.94; *p* < 0.01) and higher HR (*β* = 1.33; *p* < 0.01) (Table 9).

**TABLE 7:** Differences in foraging and control site characteristics during the brooding period of the SSME.

Variables	Foraging	Control	Statistics			*p*
			*t*	*Z*	*χ* ^*2*^	
TS (*n*)	14.22 ± 3.20	13.86 ± 2.29	0.45			0.65
TT (*n*)	140.68 ± 17.64	143.89 ± 16.67		0.70		0.49
TH (m)	37.20 ± 6.61	34.68 ± 6.08	1.48			0.15
MDBH (cm)	46.11 ± 7.36	43.05 ± 6.94	1.60			0.12
SH (m)	2.61 ± 0.57	2.54 ± 0.56	0.47			0.64
SD (%)	32.53 ± 6.21	28.44 ± 4.91		1.39		0.04*
HH (cm)	28.99 ± 8.17	30.08 ± 6.43	0.56			0.58
HCC (%)	32.13 ± 8.20	28.75 ± 8.30	1.53			0.13
C (D)	1.96 ± 1.00	2.18 ± 1.16			0.74	0.46
DR (m)	1558.80 ± 637.07	2130.43 ± 1030.92	2.50			0.02*
HR (%)	10.80 ± 3.47	5.86 ± 1.69	6.77			< 0.01**
BR (%)	16.08 ± 5.75	9.27 ± 3.85	5.21			< 0.01**
DB (m)	3435.82 ± 621.20	1839.29 ± 310.36		12.17		< 0.01**
DPNS (m)	1947.45 ± 614.21	1942.49 ± 607.44	0.03			0.98
RD (m)	2.78 ± 0.71	1.01 ± 0.30	12.06			< 0.01**
RW (m)	52.08 ± 10.21	30.16 ± 15.12		6.36		< 0.01**
WV (m/s)	0.15 ± 0.07	0.49 ± 0.09	16.08			< 0.01**
T (NTU)	2.51 ± 0.58	5.16 ± 1.16	10.87			< 0.01**

*Note:* ** represents *p* < 0.01.

**TABLE 8:** Results of AIC‐based model selection influencing foraging and control sites during the brooding period of SSME.

No	Model	df	Loglik	AICc	△AICc	ωi
1	HR + BR	2	−55.79	41.46	0.00	0.28
2	HR + DB + RW	4	−57.91	41.71	0.25	0.23
3	WV + BR + T	5	−56.42	42.07	0.61	0.18
4	BR + RD + DR	4	−56.81	42.70	1.24	0.15
5	RW + BR + SD	3	−60.33	43.02	1.56	0.06
6	T + DB	3	−67.52	43.58	1.82	0.05
7	WV + RW + DR + T	6	−62.61	43.81	1.95	0.05

**TABLE 9:** Key factors influencing foraging site selection during the brooding period of SSME after model averaging.

Parameter	Estimate	*p*	95% confidence interval
SD	0.32	0.68	0.014 to 0.603
DB	0.35	0.07	0.024 to 0.699
BR	0.94	0.01**	0.212 to 1.631
DR	0.18	0.29	0.018 to 0.305
HR	1.33	< 0.01**	0.471 to 1.993
RD	0.28	0.08	0.082 to 0.421
RW	0.46	0.15	0.094 to 0.768
WV	0.14	0.08	0.012 to 0.259
T	0.21	0.13	0.044 to 0.368

*Note:* ** represents *p* < 0.01.

## 4 Discussion

During the egg‐laying period, SSME preferred foraging sites with high concealment, a preference consistent with many bird species (e.g., *Perdix perdix* ‐Hildén 1965; *Turdus pilaris‐*Schmidt 1999). High concealment provides security by reducing disturbances and making it more difficult for predators to detect (Quinn et al. 2006; Jia 2021; Yang et al. 2024). Given that SSME migrates long distances from wintering to breeding sites, secure foraging habitats are crucial for replenishing energy reserves (Xu 2023). This preference for concealed sites may represent a trade‐off between survival and reproduction, balancing the need for safety with energy requirements necessary for successful mating (Tong et al. 2021).

During the incubation period, SSME preferred foraging sites that were farther from buildings and closer to potential nest sites. This behavior likely reflects a strategy to optimize incubation conditions by minimizing human disturbance while maintaining efficient foraging. The distance from buildings can serve as an indicator of human disturbance, which may negatively affect bird behavior and increase stress (Poudel et al. 2016; Exposito‐Granados et al. 2020). By foraging away from human activity, SSMEs minimize disruptions that could interfere with incubation. Birds, including SSMEs, often perceive human presence as a potential threat or predator, which leads them to select foraging sites farther from human disturbance (Frid and Dill 2002; Beale and Monaghan 2004; Banerjee et al. 2020). Similarly, White‐naped Cranes ( *Grus vipio* ) in Zalong Wetland also prefer less disturbed sites during incubation (Wu and Zou 2009). Minimizing exposure to human disturbance helps reduce stress levels in birds and can enhance their overall breeding success. In this study area, the onset of agricultural activities in late April likely increased disturbance, prompting SSMEs to forage farther from human settlements. Furthermore, by selecting foraging sites near their nests, SSMEs reduce travel distance, conserve energy, and maintain optimal egg temperature. This proximity allows for quick returns to the nest, improving incubation success and increasing the likelihood of healthy offspring (Erb et al. 2012).

During the brooding period, SSMEs prefer foraging sites with a high borderland ratio (BR) and heartland ratio (HR), as these areas provide optimal brood‐rearing. This preference enhances offspring survival by offering safe, energy‐conserving resting places for ducklings, which have high energetic needs and limited defenses against predators (Wooley Jr. and Owen Jr. 1978; Xue et al. 2017). By selecting sites with high BR and HR, SSMEs minimize energy expenditure, thereby improving their ability to balance both foraging and brood care (Zeng, Zhang, et al. 2015). A similar pattern was observed in the Eurasian Spoonbill ( *Platalea leucorodia* ), which also selected high HR areas for resting nestlings (Li 2017). This strategic site selection enhances reproductive success by creating safer, more efficient environments for ducklings.

Additionally, water depth plays a crucial role for piscivorous waterbirds like the SSME, influencing both foraging efficiency and habitat quality (Zeng et al. 2018). SSMEs prefer sites with greater depths and lower water velocities during various breeding stages, likely because these conditions are associated with higher food availability and more favorable foraging opportunities (Harvey and Stewart 1991; Trombulak and Frissell 2000; Giraldo et al. 2017). Investigating the specific diets and nutrient requirements of SSMEs could further clarify how these foraging site preferences contribute to energy optimization and nutritional balance throughout the breeding season.

## 5 Conclusion

Our findings highlight the distinct foraging site preferences of the SSMEs across three breeding stages, offering valuable insights for their conservation and habitat management. During the egg‐laying period, SSMEs prioritize foraging sites with high concealment to reduce predation risks and enhance mating success. In the incubation period, they minimize disturbances caused by human activities. During the brooding period, foraging sites with a high borderland ratio and heartland ratio provide safe resting areas for ducklings. Therefore, to effectively support SSME populations, conservation efforts should focus on improving habitat concealment, enriching food resources, minimizing human interference, and enhancing habitat features like borderland ratio and heartland ratio. Measures such as artificial beach‐filling and the creation of suitable resting areas will benefit SSME survival. Our study offers valuable insights into managing foraging sites for SSME in the Changbai Mountain region, emphasizing the need for site‐specific conservation strategies. However, one limitation of this study is the lack of quantification of predator‐related stress at each breeding stage. Future research should assess predator threat levels by deploying infrared cameras to monitor potential predators.

## Author Contributions

**Shengxian He:** data curation (lead), formal analysis (lead), methodology (lead), writing – original draft (lead). **Wenyu Xu:** formal analysis (equal), methodology (equal), writing – review and editing (equal). **Facai Yang:** data curation (equal). **Chen Zhang:** data curation (equal). **Keping Sun:** methodology (equal), writing – review and editing (equal). **Longcheng Fan:** methodology (equal). **Longru Jin:** conceptualization (lead), funding acquisition (lead), writing – review and editing (equal). **Haitao Wang:** conceptualization (lead), funding acquisition (lead), project administration (lead), writing – review and editing (equal).

## Conflicts of Interest

The authors declare no conflicts of interest.

## Supporting information


**Data S1.** As “Raw data and R code”. We have also provided the following captions for Sheet 1 and Sheet 2:
(1) Sheet 1: Raw data on ecological factors affecting foraging site selection at different breeding stages of the Scaly‐sided Merganser (SSME).
(2) Sheet 2: R code and results of model selection and parameter estimation.

## Data Availability

All data in this study are included as Supporting Information files.
